# Apparent reduction in heart rate during oviposition revealed by non-invasive heart rate monitoring of gravid loggerhead turtles

**DOI:** 10.3389/fphys.2025.1540252

**Published:** 2025-07-02

**Authors:** Tomoko Narazaki, Masanori Mori, Yoshimasa Matsuzawa, Ayaka Saito, Chihiro Kinoshita, Masanori Kurita, Kensuke Matsumiya, Hikari Okada, Kentaro Q. Sakamoto

**Affiliations:** ^1^ Faculty of Agriculture, Meijo University, Aichi, Japan; ^2^ Port of Nagoya Public Aquarium, Nagoya Port Foundation, Aichi, Japan; ^3^ Sea Turtle Association of Japan, Osaka, Japan; ^4^ Atmosphere and Ocean Research Institute, The University of Tokyo, Chiba, Japan

**Keywords:** heart rate, bradycardia, *Caretta caretta*, nesting, bio-logging

## Abstract

Reproductive processes place significant physiological demands on animals, often accompanied by hormonal and neural changes. In this study, we examined changes in heart rate of gravid loggerhead turtles (C*aretta caretta*) during nesting activities on the beach, especially during egg-laying phase. To examine heart rate throughout the nesting activities, non-invasive electrocardiogram (ECG) loggers and accelerometers were deployed on five gravid females. Heart rate increased markedly upon beach landing and remained elevated during most nesting phases. However, a significant decrease in heart rate, often accompanied by increased RMSSD, was observed during egg-laying, suggesting parasympathetic nervous system dominance during this phase. This pattern is similar to observation reported in other species (e.g., horses and chum salmon), where bradycardia during reproductive events is associated with elevated parasympathetic tone. Our study reported an apparent reduction in heart rate during oviposition, which reflects the physiological mechanisms underlying nesting activities in sea turtles, and suggest that external stressors disrupting parasympathetic activity may reduce egg-laying success.

## 1 Introduction

Heart rate, an important physiological parameter reflecting an animal’s internal state, is widely used in studies to estimate energy expenditure (Butler et al., 2002; Weimerskirch et al., 2002), evaluate stress and health conditions (Ellenberg et al., 2006; von Borell et al., 2007), and understand adaptations to physiologically challenging environments such as hypoxia during breath-hold diving and high-altitude flights (Ponganis et al., 1997; Wright et al., 2014; Meir et al., 2019; Saito et al., 2024). Reproductive processes (e.g., egg-laying, parturition) are essential biological processes for species survival. However, these processes place significant physiological demands on the reproductive animal, often accompanied by hormonal changes and physical exertion. Monitoring heart rate and heart rate variability, which serve as indicators of autonomic nervous system activity, has become an important tool for studying the drastic changes in maternal condition during reproductive processes of livestock (Nagel et al., 2014; Kovács et al., 2015). For example, in horses, parturition is associated with a decrease in heart rate, and the occurrence of arrhythmia was observed during the 15 min prior to and 45 min following delivery (Nagel et al., 2014). Analysis of heart rate variability and plasma catecholamine concentration patterns suggest that high parasympathetic activity is a prerequisite for the onset of parturition, with horses giving birth in a state of marked relaxation and elevated parasympathetic tone (Nagel et al., 2014). Similarly, in cows, an increase in the high-frequency component of heart rate variability, which reflects parasympathetic activity, is observed following the onset of behavioral signs of parturition (Kovács et al., 2015). Heart rate and heart rate variability indicators are also used to improve the prediction of the time of calving, which is crucial for maintaining efficient and profitable daily farming (Kishi et al., 2024). In contrast, studies on heart rate during reproductive process in non-livestock animals are scarce, particularly for marine animals (but see Wells, 1979 for octopus; Makiguchi et al., 2009 for chum salmon).

Sea turtles spend most of their lives in the ocean; however, they emerge onto beaches to nest. The nesting process typically involves crawling up the beach, digging a body pit and egg chamber, laying eggs, covering the nest, and returning to the sea (Miller et al., 1997). These terrestrial nesting activities place significant physical and physiological demands on turtles (Jackson and Prange, 1979). For example, green turtles are shown to experience a tenfold increase in metabolic rate compared to resting levels during nesting activities (Jackson and Prange, 1979). Numerous hormonal studies have provided valuable insights into general hormone patterns (Owens, 1997; Jessop and Hamann, 2004); however, little is known about the changes in heart rate and neural activity associated with nesting behaviors.

Nesting turtles on the beach are known to be sensitive and often halt their nesting behavior in response to external disturbances. However, once egg-laying begins, the turtle generally continues laying eggs, even in the presence of disturbances (Miller et al., 1997). Based on this observation, we hypothesized that high parasympathetic nervous system activity may play a role in sea turtle egg-laying, as has been reported in horse parturition (Nagel et al., 2014). Parasympathetic activity is typically dominant during relaxed states and helps lower heart rate through the vagus nerve. To test this hypothesis, we monitored the temporal changes in heart rate of minimally disturbed loggerhead turtles (*Caretta caretta*) throughout their nesting activities using a non-invasive electrocardiogram data logger (Sakamoto et al., 2021). If supported, the hypothesis predicts a decrease in heart rate during egg-laying.

## 2 Materials and methods

### 2.1 Animals and study sites

All experimental procedures were covered by the guidelines of the Animal Ethics Committee of Meijo University, and the protocol of the study was approved by this committee (2021A13, 2022A17, 2023A14, 2023A15, 2024A23). Electrocardiogram (ECG) data from gravid female loggerhead turtles were collected throughout their nesting activities using animal-borne ECG loggers at two locations in Japan: Port of Nagoya Public Aquarium (35°5’N, 136°52’E) and Senri beach in Minabe, Wakayama (33°46’N, 135°18’E). At the aquarium, the measurements were conducted in 2021 and 2023 on captive turtles whose follicle development had been confirmed via ultrasound examination. The turtles equipped with loggers were kept in a tank connected to an artificial beach, allowing them to move freely between the tank and the beach. Nesting behavior was monitored with three night-vision cameras installed at the artificial beach. Once oviposition was confirmed, the data loggers were retrieved from the turtles the following morning. The field study in Minabe was conducted during the 2022, 2023 and 2024 nesting seasons. Once the turtles emerged onto the nesting beach, the turtles were promptly captured, fitted with data loggers, and released at the same site. When the turtles were returned on the beach, their nesting behavior was observed from a distance to avoid disturbance. After oviposition was confirmed, the turtles were recaptured just before they return to the sea to retrieve the data loggers.

### 2.2 Data loggers

The ECG was recorded at 250 Hz (ECG400-DT; Little Leonardo, Tokyo, Japan; 21 mm width, 64 mm length, 23 mm height, 60 g mass in air) using a non-invasive method by attaching two electrode pads to the turtles’ carapace, following Sakamoto et al. (2021) with some modifications (Kinoshita et al., 2022). A step-by-step instruction with detailed illustrations for attaching the electrodes and loggers is provided in Kinoshita et al. (2022). While electrode pads on the plastron provide stronger ECG signals (Kinoshita et al., 2022), the pads were attached to the carapace due to the high risk of detachment while the turtles crawl on the beach. The activity of the turtles was recorded using a behavioral logger: a M190L-D2GT (Little Leonardo; cylindrical shape; 15 mm diameter, 53 mm length, 18 g mass in air) recording 2-axis acceleration, depth and temperature, or a W2000-3MPD3GT (Little Leonardo; cylindrical shape; 26 mm diameter, 175 mm length, 140 g mass in air) recording 3-axis acceleration and the Earth’s magnetic field, depth, and temperature. Acceleration was recorded at either 16 Hz (CcW19 and M3) or 32 Hz (CcW06, M4 and M7). Depth, temperature and the Earth’s magnetic field were recorded at 1 Hz.

### 2.3 Data analysis

**FIGURE 1:**
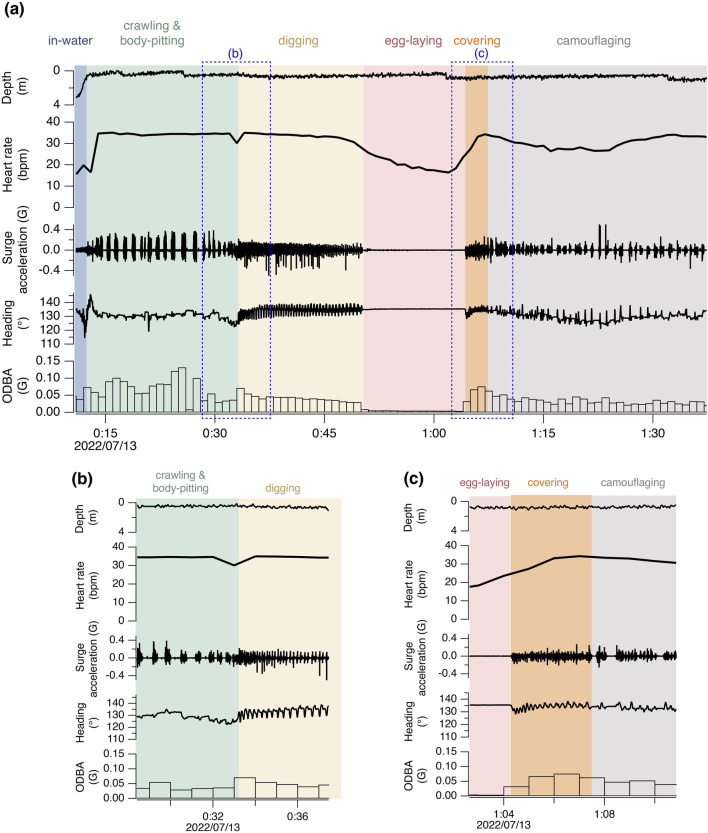
Time-series data of heart rate, depth, and high-frequency surge acceleration, heading, and 1-minute mean ODBA during the nesting activity of turtle M3. The areas enclosed by the blue dashed lines in **(a)** are magnified in **(b, c)**.

Nesting activities were categorized into 5 phases based on visual observation of the captive turtles and acceleration-magnetometer recordings of wild turtles: 1) crawling and body-pitting, 2) digging an egg chamber, 3) laying eggs, 4) covering the egg chamber, and 5) camouflaging the site (Figure 1; Supplementary Figure S1). While crawling and body-pitting are typically classified separately, they were grouped into the same category in this study due to difficulty in distinguish between them based on acceleration-magnetometer recordings. The definitions of classifications are provided in Supplementary Material.

Time-series biologging data were analyzed using IGOR Pro version 8.04 (Wavemetrics, Portland, OR, United States). To remove noise primarily caused by turtles’ movements, the ECG recordings were processed using the ECGtoHR program, which employs an FIR filter with a Hanning window to isolate the QRS complex and detect R peaks (Sakamoto et al., 2021). The parameters of the ECGtoHR program were visually adjusted to set a QRS frequency of 10–12 Hz and maximum heart rate of 60–80 bpm for each deployment (Sakamoto et al., 2021). The instantaneous heart rate was then calculated as the reciprocal of the time interval between consecutive R waves (RR interval). The heart rate was calculated as the median of instantaneous heart rate per minute. In addition, the root mean square of successive differences in consecutive RR intervals (RMSSD) was quantified every minute. RMSSD is a widely used time-domain measure of heart rate variability, with higher values indicating greater parasympathetic nervous system activity (Shaffer et al., 2014).

The acceleration data recorded by behavioral loggers was separated into two components using a frequency-based filter: a high-frequency dynamic body acceleration component (i.e., flipper movements) and a low-frequency gravity-based acceleration component (Shiomi et al., 2010). As a proxy for activity level, overall dynamic body acceleration (ODBA) in G was calculated by summing the absolute values of dynamic acceleration at 16 or 32 Hz across the three axes of the W2000-3MPD3GT (Wilson et al., 2006). ODBA was not calculated for the turtle (i.e., CcW06) equipped with the M190L-D2GT. The orientation of turtles (pitch, roll and heading) was calculated every second using 3-axis magnetism and low-frequency acceleration data using the macro ThreeD_path, which is compatible with IGOR Pro (Shiomi et al., 2010; https://japan-biologgingsci.org/home/macro/threed_path/).

Variations in heart rate throughout the nesting activities were examined using a linear mixed model (LMM). As data obtained from the same nesting event was not independent, ‘nesting ID’ was included as random effect. Explanatory variables were the behavioral modes (i.e., crawling, digging, egg-laying, covering the egg chamber, camouflaging) and ODBA. The most parsimonious model was selected based on AIC, followed by the Tukey-Kramer *post hoc* test for the behavioral modes. All statistical analysis was performed using R (The R project for Statistical Computing, http://www.r-project.org).

## 3 Results

ECG and behavioral data were collected from a total of five gravid sea turtles over seven nesting events, including a pseudo-nesting event by turtle M7 (Table 1). The wild turtles equipped with loggers returned to nest the following night (M3, 4) or the second night after release (M7). M7 exhibited behavior consistent with normal nesting: it created a body pit, dug an egg chamber, and displayed typical egg-laying behaviors, such as letting its tail hang into the egg chamber and remained motionless for a while. However, the only difference from normal nesting was that no eggs were actually laid. After completing this pseudo-egg-laying, M7 filled the chamber and camouflaged the site.

**TABLE 1:** Descriptive statistics during nesting activity and egg-laying phase.

Turtle ID	Study site^a^	Accelerometer type	Time of landing	Water temperature (°C)	Temperature at the beach (°C)	Whole period on the beach^b^		Egg-laying phase	
						Duration (min)	Heart rate (bpm)	Duration (min)	Heart rate (bpm)
CcW06	PNPA	D2GT^c^	2021/05/16 21:37	26.7 ± 0.04	24.5 ± 0.5	211	27.6 ± 5.6	15.7	17.7 ± 5.2
		D2GT	2021/06/09 22:56	27.9 ± 0.03	26.7 ± 0.3	174	29.1 ± 5.2	10.6	21.6 ± 6.1
CcW19	PNPA	3MPD3GT	2023/04/30 02:26	25.7 ± 0.1	23.9 ± 0.2	162	28.2 ± 5.2	29.1	19.2 ± 4.5
		3MPD3GT	2023/05/12 21:57	26.7 ± 0.1	25.8 ± 0.5	226	24.2 ± 4.5	30.9	16.0 ± 2.9
M3	M	3MPD3GT	2022/07/13 00:12	27.2 ± 0.3	25.8 ± 0.5	94	30.4 ± 5.3	14.1	20.7 ± 3.6
M4	M	3MPD3GT	2023/07/07 22:31	24.3 ± 0.6	26.1 ± 0.2	103	27.2 ± 9.6	25.0	12.5 ± 5.7
M7^d^	M	3MPD3GT	2024/06/24 22:33	24.2 ± 0.09	23.7 ± 0.1	75	24.3 ± 7.6	11.8	11.6 ± 3.5

Mean heart rate, temperature and heart rate are provided with standard deviation. Water temperatures during the 90 min before landing are presented.

^a^ The abbreviations of the study sites are PNPA for Port of Nagoya Public Aquarium and M for Minabe.

^b^ From beach landing to return to the pool for PNPA turtles; from beach landing to logger retrieval during camouflage phase for Minabe turtles.

^c^ No data due to logger failure.

^d^ The duration and heart rate during a pseudo-nesting behavior are presented for M7.

**FIGURE 2:**
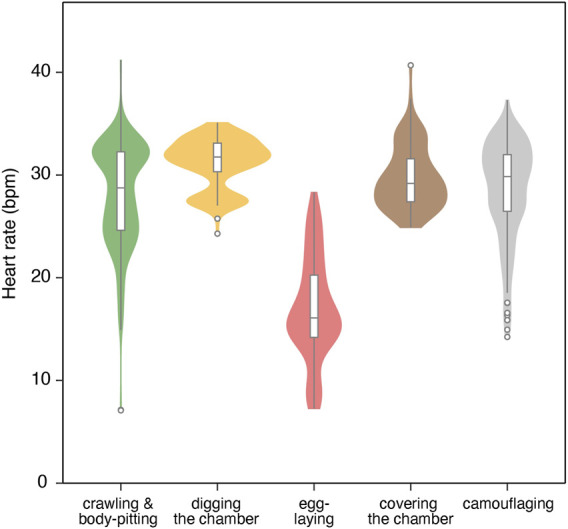
Violin/box plot for heart rate across different behavioral modes of four turtles during six nesting events. The box represents the interquartile range (IQR), with outliers (beyond 1.5 x IQR) shown as points beyond the whiskers.

**FIGURE 3:**
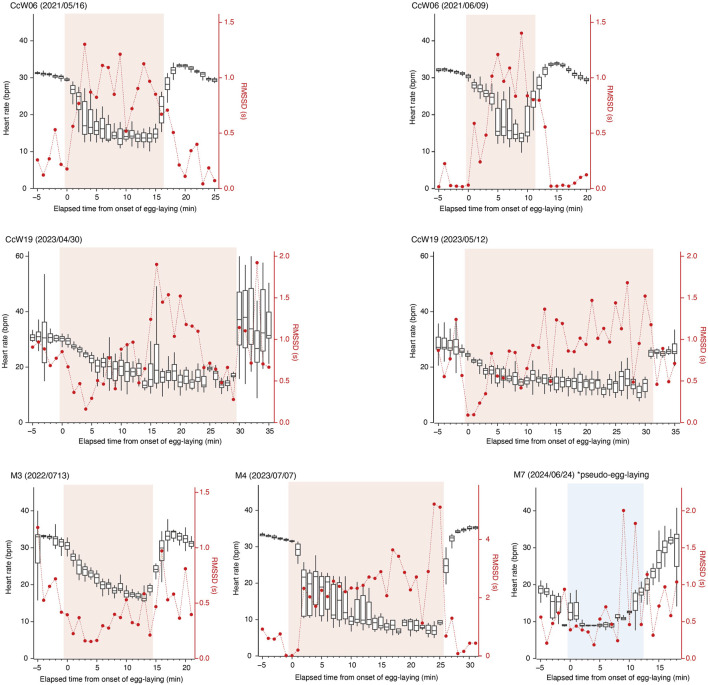
Temporal changes in heart rate (box plots) and RMSSD (red dots) before, during, and after the egg-laying periods. RMSSD and the median heart rate are shown for each minute. The upper and lower edges of the box represent the third and first quartiles, respectively. Time zero corresponds to the onset of the egg-laying period. The egg-laying and pseudo-egg-laying periods are highlighted in pink and blue, respectively.

For the six true nesting events, the heart rate during the 10 min preceding beach landing was 19.7 ± 7.0 bpm (mean ± s.d.), but it rapidly increased to 29.3 ± 5.5 bpm during the 10 min immediately after landing (Figure 1; Supplementary Figure S1). There was substantial variability in heart rate during nesting events, with a weak positive relationship observed with ODBA (P < 0.05, F (1, 576.69) = 20.4, Supplementary Figure S2). The heart rate during egg-laying was significantly lower than during any other behavioral modes (P < 0.05, F (4, 576.91) = 138.6; 16.9 ± 5.0 bpm, Figure 2), showing a 54% decrease compared to the heart rate during the preceding digging the egg chamber (31.2 ± 2.4 bpm). With the onset of egg-laying, the heart rate gradually decreased, accompanied by increased variability (Figure 3). This decrease in heart rate was not only due to the increase in RR intervals but also associated with greater variability in the RR intervals: the mean RR intervals during the 5 min prior to egg-laying were 2.0 ± 0.5 s, increasing to 3.5 ± 1.5 s during egg-laying. A general trend of increase in RMSSD was also observed during egg-laying (Figure 3). After the completion of egg-laying, the heart rate rapidly returned to levels comparable to those measured prior to egg-laying (Figure 3).

During pseudo-egg-laying observed in M7, a decrease in heart rate was noted at the onset of the pseudo-egg-laying. However, the return of heart rate after the completion of pseudo-egg-laying was more gradual to the return observed after true egg-laying (Figure 3).

## 4 Discussion

By using non-invasive ECG monitoring method, we were able to measure heart rate in loggerhead turtles throughout nesting activities. In all turtles, a marked increase in heart rate was recorded upon landing on the beach. Loggerhead turtles have been reported to exhibit diving bradycardia as an adaptation to reduce oxygen consumption during breath-holding dives (Saito et al., 2024). It is likely that a similar bradycardic response occurred until their bodies were fully out of the water, although apnea occurs both in water and on land. Once on the beach, the heart rate generally remained elevated at 24.2–30.4 bpm, although it varied depending on the behavioral modes (Table 1; Figure 1). In diving sea turtles, an increase in heart rate corresponding to underwater activity intensity has been reported in captive turtles (Williams et al., 2019; Okuyama et al., 2020), as oxygen must be transported from their primary oxygen store in the lungs to the muscles (Lutcavage et al., 1997). In nesting turtles on the beach, a positive relationship between heart rate and activity intensity was also observed, with the relationship being weak (Supplementary Figure S2). Nesting activities on the beach are considered physically and physiologically demanding (Jackson and Prange, 1979). Furthermore, the lower specific heat capacity of air compared to water facilitates an increase in body temperature, which in turn raises metabolic rate, although the effect of temperature may be small in this study due to relatively low air temperature (Table 1). One possible reason for the weak relationship between heart rate and activity intensity is that the heart rate while on the beach may already be at very high level for sea turtles, leaving little capacity for significant increase in response to high exercise intensity. In fact, the heart rates recorded in this study represent the highest levels reported for loggerhead turtles, even exceeding those observed during inter-dive surface intervals when heart rate typically increases (Saito et al., 2022; 2024; Williams et al., 2019). Another possibility could be that turtles may be exposed to other factors (e.g., external stimuli, internal condition associated with egg-laying) that influence heart rate aside from activity intensity. Similarly, the relationship between heart rate and activity observed in turtles freely diving in their natural environment was weaker than that reported during voluntary diving in a simpler captive environment (Saito et al., 2024).

Our results showed a significant decrease in heart rate associated with egg-laying, as predicted by the hypothesis that egg-laying is linked to parasympathetic nervous system activity. Upon the onset of egg-laying, a gradual decrease in heart rate was observed, resulting from an increased RR interval and its variability, suggesting the potential occurrence of arrhythmia (Figures 1, 3). This pattern in heart rate is similar to that observed during horse parturition, which occurs under elevated parasympathetic tone (Nagel et al., 2014). Similar phenomena have also been reported in non-mammalian species: transient cardiac arrest, considered an extreme case of bradycardia, was observed at the moment of gamete transfer and release in octopus (*Octopus vulgaris*) and chum salmon (*Oncorhynchus keta*), respectively (Wells, 1979; Makiguchi et al., 2009). The decrease in heart rate can result from increased parasympathetic nervous activity and/or decreased sympathetic activity. However, the low norepinephrine levels observed during arrhythmia events in horses (Nagel et al., 2014) and the abolition of cardiac arrest following administration of anticholinergic drugs in chum salmon (Makiguchi et al., 2009) indicate that these reproductive processes occurred under a high parasympathetic tone. In this study, a general trend of increased RMSSD was observed in association with bradycardia during egg-laying, suggesting that parasympathetic nervous activity may play a dominant role in this process. In contrast to the gradual decrease in heart rate at the onset of egg-laying, the sharp return to baseline levels after the completion of egg-laying suggests that different mechanisms may be involved in controlling heart rate (Figure 3). Interestingly, a similar decrease in heart rate was observed in M7 during pseudo-egg-laying, although the temporal pattern of RMSSD was different, remaining relatively low. Additionally, the recovery of heart rate to the baseline was more gradual toward the end of pseudo-egg-laying. The only observed difference between pseudo- and true egg-laying was the absence of actual oviposition. It is possible that the rapid recovery of heart rate may be triggered by physiological changes, such as hormonal shifts and neural activity, associated with the successful completion of egg-laying. Further studies, including hormonal and/or pharmacological researches, to examine autonomic nervous system control, are important for elucidating physiological mechanism underlying nesting activities.

This study represents the first heart rate monitoring of sea turtles during nesting activities, demonstrating that heart rate remained elevated throughout most of the nesting activities, except during egg-laying. This indicates that nesting turtles have high metabolic rate, likely driven by physical demands, such as supporting their body weight, and increased body temperature. Considering that nesting activities on the beach typically last for over an hour, these results suggest high physiological strain associated with the activity. Furthermore, our data suggest that egg-laying in sea turtles occurs under parasympathetic tone, although further investigation into hormonal patterns and heart rate variability is necessary for validation. The parasympathetic nervous system is in an antagonistic relationship with the stress-activated sympathetic nervous system. If high parasympathetic activity is a prerequisite for initiating egg-laying, external stressors, such as disturbance from tourists or strong light, may significantly reduce egg-laying success. Further studies uncovering the physiological mechanisms underlying nesting activities with ecological perspectives could provide valuable insights for the effective conservation and management of endangered sea turtles.

## Data Availability

The original contributions presented in the study are included in the article/Supplementary Material, further inquiries can be directed to the corresponding author.

## References

[B1] Butler P. J. Frappell P. B. Wang T. Wikelski M. (2002). The relationship between heart rate of oxygen consumption in Galapagos marine iguanas (*Amblyrhynchus cristatus*) at two different temperatures. J. Exp. Biol. 205, 1917–1924. 10.1242/jeb.205.13.1917 12077168

[B2] Ellenberg U. Mattern T. Seddon P. J. Jorquera G. L. (2006). Physiological and reproductive consequences of human disturbance in humboldt penguins: the need for species-specific visitor management. Biol. Conserv. 133, 95–106. 10.1016/j.biocon.2006.05.019

[B3] Jackson D. C. Prange H. D. (1979). Ventilation and gas exchange during rest and exercise in adult green sea turtles. J. Comp. Physiol. 134, 315–319. 10.1007/bf00709998

[B4] Jessop T. S. Hamann M. (2004). Hormonal and metabolic responses to nesting activities in the green turtle, *Chelonia mydas* . J. Exp. Mar. Biol. Ecol. 308, 253–267. 10.1016/j.jembe.2004.03.005

[B5] Kinoshita C. Saito A. Kawai M. Sato K. Sakamoto K. Q. (2022). A non-invasive heart rate measurement method is improved by placing the electrodes on the ventral side rather than the dorsal in loggerhead turtles. Front. Physiol. 13, 811947. 10.3389/fphys.2022.811947 35250617 PMC8889138

[B6] Kishi S. Kojima T. Huang C. Yayou K. Fujioka K. (2024). A feasibility study on predicting cow calving time over 40h in advance using heart rate and financial technical indicators. Sci. Rep. 14, 21748. 10.1038/s41598-024-72521-w 39294265 PMC11411094

[B7] Kovács L. Tőzsér J. Kézér F. L. Ruff R. Aubin-Wodala M. Albert E. (2015). Heart rate and heart rate variability in multiparous dairy cows with unassisted calvings in the periparturient period. Physiol. Behav. 129, 281–289. 10.1016/j.physbeh.2014.11.039 25449409

[B8] Lutcavage M. E. Lutz P. L. (1997). “Diving physiology,” in The biology of sea turtles (Boca Raton, FL: CRC Press), 277–296.

[B9] Makiguchi Y. Nagata S. Kojima T. Ichimura M. Konno Y. Murata H. (2009). Cardiac arrest during gamete release in chum salmon regulated by the parasympathetic nerve system. PLoS ONE 4 (6), e5993. 10.1371/journal.pone.0005993 19543389 PMC2694361

[B10] Meir J. U. York J. M. Chua B. A. Jardine W. Hawkes L. A. Milsom W. K. (2019). Reduced metabolism supports hypoxic flight in the high-flying bar-headed goose (*Anser indicus*). eLife 8, e44986. 10.7554/eLife.44986 31478481 PMC6721836

[B11] Miller J. D. (1997). “Reproduction in sea turtles,” in The biology of sea turtles (Boca Raton, FL: CRC Press), 51–81.

[B12] Nagel C. Erber R. Ille N. von Lewinski M. Aurich J. Möstl E. (2014). Parturition in horses is dominated by parasympathetic activity of the autonomous nervous system. Theriogenology 82, 160–168. 10.1016/j.theriogenology.2014.03.015 24767599

[B13] Okuyama J. Shiozawa M. Shioide D. (2020). Heart rate and cardiac response to exercise during voluntary dives in captive sea turtles (cheloniidae). Biol. Open 9, bio049247. 10.1242/bio.049247 32033966 PMC7055368

[B14] Owens D. W. (1997). “Hormones in the life history of sea turtles,” in The biology of sea turtles. Editors Lutz P. L. Musick J. A. (Boca Raton, FL: CRC Press), 315–342.

[B15] Ponganis P. J. Kooyman G. L. Winter L. M. Starke L. N. (1997). Heart rate and plasma lactate responses during submerged swimming and trained diving in California sea lions, *Zalophus californianus* . J. Comp. Physiol. B 167, 9–16. 10.1007/s003600050042 9051904

[B16] Saito A. Kinoshita C. Kawai M. Fukuka T. Sato K. Sakamoto K. Q. (2022). Effects of a parasympathetic blocker on the heart rate of loggerhead sea turtles during voluntary diving. J. Exp. Biol. 225, jeb243922. 10.1242/jeb243922 35441228

[B17] Saito A. Kinoshita C. Sakai K. Sato K. Sakamoto K. Q. (2024). Heart rate reduction during voluntary deep diving in free-ranging loggerhead sea turtles. J. Exp. Biol. 227, jeb246334. 10.1242/jeb.246334 38442390 PMC10949068

[B18] Sakamoto K. Q. Miyayama M. Kinoshita C. Fukuoka T. Ishihara T. Sato K. (2021). A non-invasive system to measure heart rate in hard-shelled sea turtles: potential for field applications. Phil. Trans. R. Soc. B 376, 20200222. 10.1098/rstb.2020.0222 34121465 PMC8200654

[B19] Shaffer F. McCraty R. Zerr C. L. (2014). A healthy heart is not a metronome: an integrative review of the heart’s anatomy and heart rate variability. Front. Psychol. 5, 1040. 10.3389/fpsyg.2014.01040 25324790 PMC4179748

[B20] Shiomi K. Narazaki T. Sato K. Shimatani K. Arai N. Ponganis P. J. (2010). Data-processing artefacts in three-dimensional dive path reconstruction from geomagnetic and acceleration data. Aquat. Biol. 8, 289–294. 10.3354/ab00239

[B21] von Borell E. Langbein J. Després G. Hansen S. Leterrier C. Marchant J. (2007). Heart rate variability as a measure of autonomic regulation of cardiac activity for assessing stress and welfare in farm animals - a review. Physiol. Behav. 92 (3), 293–316. 10.1016/j.physbeh.2007.01.007 17320122

[B22] Weimerskirch H. Shaffer S. A. Mabille G. Martin J. Boutard O. Rouanet J. L. (2002). Heart rate and energy expenditure of incubating wandering albatrosses: basal levels, natural variation, and the effects of human disturbance. J. Exp. Biol. 205, 475–483. 10.1242/jeb.205.4.475 11893761

[B23] Wells M. J. (1979). The heartbeat of *Octopus vulgaris* . J. Exp. Biol. 78, 87–104. 10.1242/jeb.78.1.87

[B24] Williams C. L. Sato K. Ponganis P. J. (2019). Activity, not submergence, explains diving heart rates of captive loggerhead sea turtles. J. Exp. Biol. 222, jeb200824. 10.1242/jeb.200824 30936271

[B25] Wilson R. White C. Quintana F. Halsey L. Liebsch N. Martin G. (2006). Moving towards acceleration for estimates of activity-specific metabolic rate in free-living animals: the case of the cormorant. J. Anim. Ecol. 75, 1081–1090. 10.1111/j.1365-2656.2006.01127.x 16922843

[B26] Wright A. K. Ponganis K. V. McDonald B. I. Ponganis P. J. (2014). Heart rates of emperor penguins diving at sea: implications for oxygen Store management. Mar. Ecol. Prog. Ser. 496, 85–98. 10.3354/meps10592

