# Supplementary material for: Apparent reduction in heart rate during oviposition revealed by non-invasive heart rate monitoring of gravid loggerhead turtles
Source: Front Physiol. 2025 Jul 2;16:1540252. doi: 10.3389/fphys.2025.1540252 (PMC12263587; doi:10.3389/fphys.2025.1540252)
Supplement: Supplementary file 2 [file DataSheet1.docx]

**Supplementary materials**

**Definition of behavioral classification**

In this study, the period from when the turtle’s body was fully out of water (depth $\leq$ 0 m) until it started digging the egg chamber was classified as ‘crawling and body-pitting’. The ‘crawling and body-pitting’ phase is characterized by distinct rhythmic peaks in the high-frequency component of surge acceleration (with cycles of tens of seconds to several minutes), which reflect their flipper movements. While crawling and body-pitting are typically classified separately, they were grouped into the same category in this study due to the difficulty of distinguishing between them based on acceleration-magnetometer recordings. After completing body-pitting, the turtle digs an egg chamber by alternately using its hindlimbs, causing the carapace to sway. Thus, the period in which rhythmic peaks appear in the high-frequency surge acceleration (with cycles of several to tens of seconds), along with periodic fluctuations in heading, was classified as ‘digging an egg chamber’. Following digging, the phase in which both acceleration and heading remain stable was classified as ‘egg-laying’. After egg-laying, the turtle uses its hindlimbs alternately to fill the egg chamber, causing rhythmic peaks in the high-frequency surge acceleration (with cycles of several to tens of seconds), similar to the digging phase, along with periodic fluctuations in heading. This period was classified as ‘filling’.　Following filling, the period in which distinct rhythmic peaks appear in the high-frequency surge acceleration (with cycles of tens of seconds to several minutes) with no specific characteristics in heading was classified as ‘camouflaging’. After completing camouflaging, the turtle crawled back to the water. The acceleration data during camouflaging and crawling exhibit similar characteristics. Therefore, for captive turtles, post-camouflaging crawling was classified based on night vision recordings. For wild turtles, no data were available for post-camouflaging crawling, as they were recaptured immediately after camouflaging to retrieve data loggers.


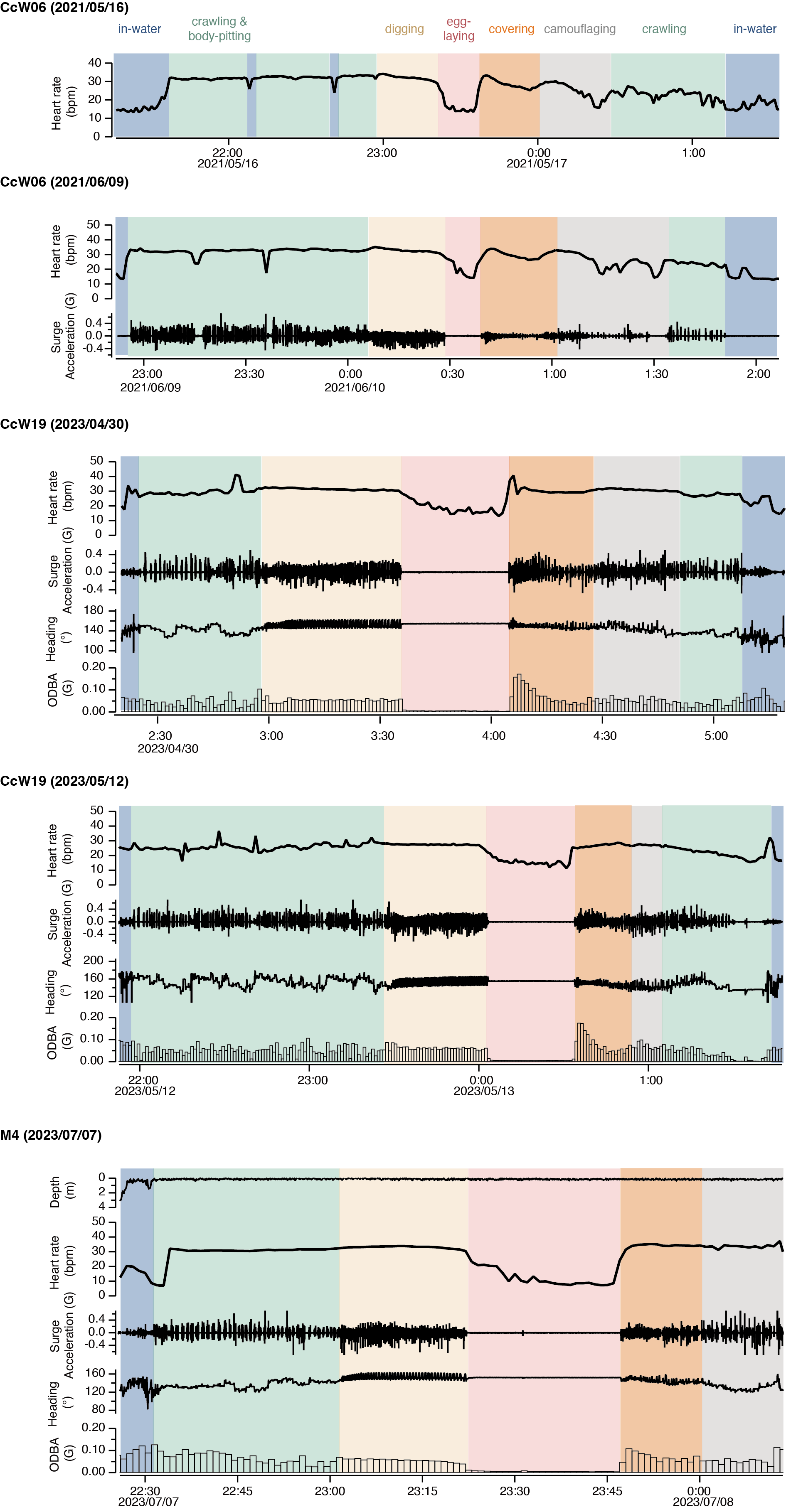


**Supplementary Figure 1.** Time-series data of heart rate and behavioral parameters during nesting activities. Depth data were not presented for captive turtles (CcW06 and CcW19) due to insufficient resolution of the depth sensor for the captive tank. Only heart rate data were available for CcW06 (2021/05/06) due to malfunction in the accelerometer. For CcW19 and M4, 1-min mean ODBA was presented.


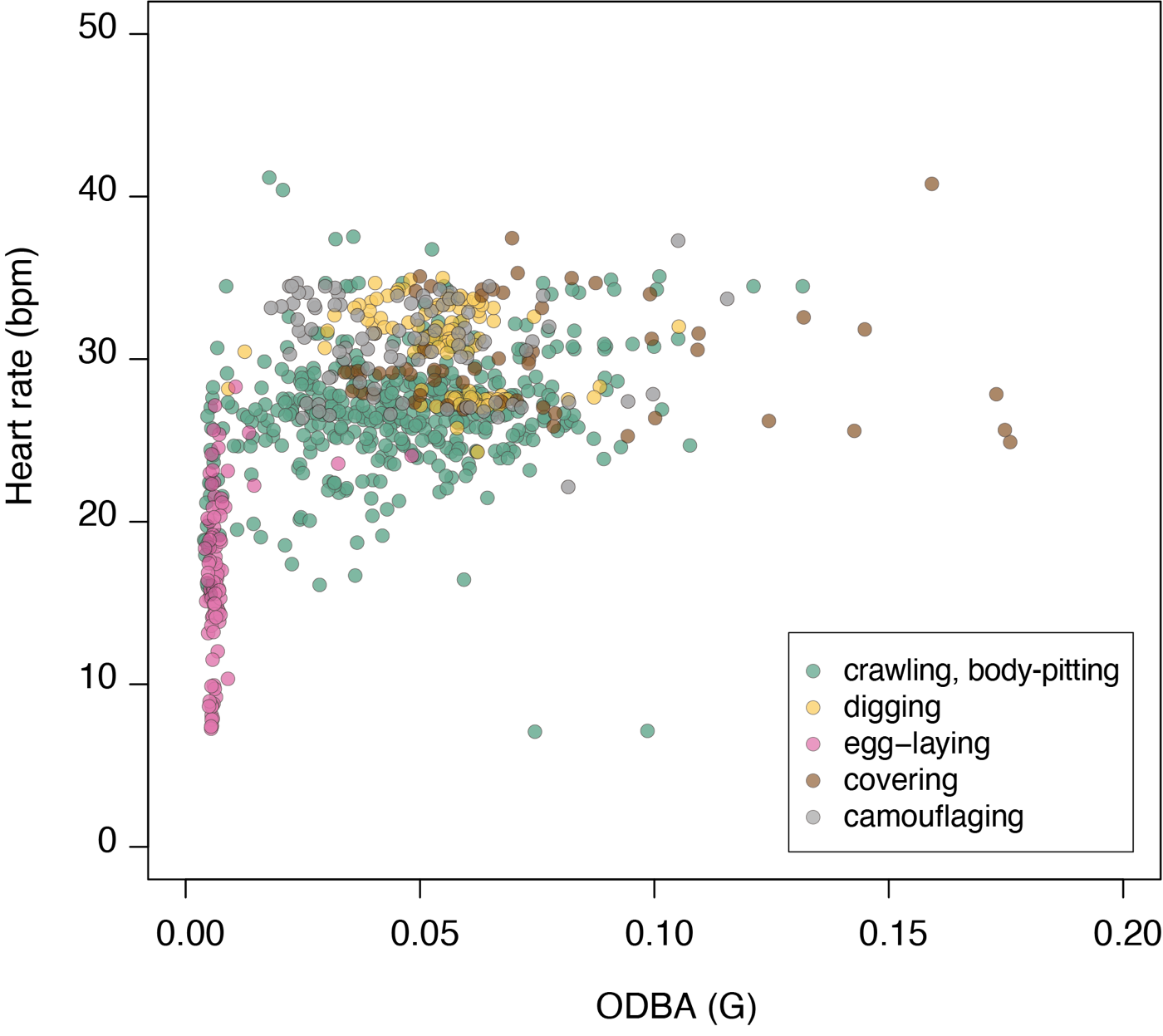


**Supplementary Figure 2.** Relationship between ODBA and heart rate during nesting activities. Data from a total six nesting events of four turtles were plotted.
